# QuEChERS-超高效液相色谱-串联质谱法同时测定水果中36种真菌毒素

**DOI:** 10.3724/SP.J.1123.2022.12010

**Published:** 2023-09-08

**Authors:** Rui ZHAO, Qingwen HUANG, Zhiying YU, Zheng HAN, Kai FAN, Zhihui ZHAO, Dongxia NIE

**Affiliations:** 1.上海海洋大学食品学院, 上海 201306; 1. College of Food Sciences and Technology, Shanghai Ocean University, Shanghai 201306, China; 2.上海市农业科学院农产品质量标准与检测技术研究所, 上海 201403; 2. Institute for Agro-Food Standards and Testing Technology, Shanghai Academy of Agricultural Sciences, Shanghai 201403, China; 3.上海师范大学生命科学学院, 上海 201418; 3. College of Life Science, Shanghai Normal University, Shanghai 201418, China

**Keywords:** QuEChERS, 超高效液相色谱-串联质谱, 真菌毒素, 水果, QuEChERS, ultra performance liquid chromatography-tandem mass spectrometry (UPLC-MS/MS), mycotoxins, fruits

## Abstract

基于QuEChERS-超高效液相色谱-串联质谱法(UPLC-MS/MS),建立了水果(草莓、葡萄、梨和桃)中36种真菌毒素的准确定量分析方法,并应用该方法对不同水果中真菌毒素的污染情况进行了分析。2.0 g样品用10 mL乙酸-乙腈-水(1∶79∶20, v/v/v)提取,无水硫酸镁(2.0 g)和氯化钠(0.5 g)盐析,离心后采用85 mg十八烷基硅烷键合硅胶(C_18_)和15 mg *N*-丙基乙二胺(PSA)对上清液进行吸附净化,浓缩复溶后过滤膜;采用Waters XBridge BEH C_18_色谱柱分离,以5 mmol/L醋酸铵水溶液和甲醇为流动相进行梯度洗脱,采用电喷雾离子源(ESI)正、负离子切换多反应监测模式测定,以空白基质匹配外标曲线法准确定量。结果表明,36种真菌毒素在8.5 min内完成色谱分离,并在各自的线性范围内具有良好的线性关系,相关系数(*R*^2^)≥0.990,检出限(LOD)和定量限(LOQ)分别为0.02~5 μg/kg和0.1~10 μg/kg。在低、中、高3个添加水平下,4种水果基质中36种真菌毒素的平均加标回收率为77.0%~118.9%,日内精密度为1.3%~14.9%,日间精密度为0.2%~17.3%。利用所建立的方法对60份水果样品(15份草莓、15份葡萄、15份梨和15份桃)进行检测,共检出11种真菌毒素,平均含量为0.13~35.85 μg/kg,其中3份样品存在多种真菌毒素共同污染的情况。本方法操作简便,快速准确,灵敏度高,重复性与稳定性良好,适用于草莓、葡萄、梨和桃中36种真菌毒素的同时检测。

## Abstract (en)

Mycotoxins are secondary metabolites produced by toxigenic fungi under specific environmental conditions. Fruits, owing to their high moisture content, rich nutrition, and improper harvest or storage conditions, are highly susceptible to various mycotoxins, such as ochratoxin A (OTA), zearalenone (ZEN), patulin (PAT), *Alternaria* toxins, etc. These mycotoxins can cause acute and chronic toxic effects (teratogenicity, mutagenicity, and carcinogenicity, etc) in animals and humans. Given the high toxicity and wide prevalence of mycotoxins, establishing an efficient analytical method to detect multiple mycotoxins simultaneously in different types of fruits is of great importance. Conventional mycotoxin detection methods rely on high performance liquid chromatography (HPLC) coupled with mass spectrometry (MS). However, fruit sample matrices contain large amounts of pigments, cellulose, and minerals, all of which dramatically impede the detection of trace mycotoxins in fruits. Therefore, the efficient enrichment and purification of multiple mycotoxins in fruit samples is crucial before instrumental analysis. In this study, a reliable method based on a QuEChERs sample preparation approach coupled with ultra performance liquid chromatography-tandem mass spectrometry (UPLC-MS/MS) was established to determine 36 mycotoxins in fruits. In the optimal extraction method, 2.0 g of a sample was extracted with 10 mL of acetic acid-acetonitrile-water (1∶79∶20, v/v/v) in a 50 mL centrifuge tube, vortexed for 30 s, and ultrasonicated for 40 min. The mixture was then salted out with 2.0 g of anhydrous MgSO_4_ and 0.5 g of NaCl and centrifuged for 5 min. Next, 6 mL of the supernatant was purified using 85 mg of octadecylsilane-bonded silica gel (C_18_) and 15 mg of *N*-propylethylenediamine (PSA). After vigorous shaking and centrifugation, the supernatant was collected and dried with nitrogen at 40 ℃. Finally, the residues were redissolved in 1 mL of 5 mmol/L ammonium acetate aqueous solution-acetonitrile (50∶50, v/v) and passed through a 0.22 μm nylon filter before analysis. The mycotoxins were separated on a Waters XBridge BEH C_18_ column using a binary gradient mixture of ammonium acetate aqueous solution and methanol. The injection volume was 3 μL. The mycotoxins were analyzed in multiple reaction monitoring (MRM) mode under both positive and negative electrospray ionization. Quantitative analysis was performed using an external standard method with matrix-matched calibration curves. Under optimal conditions, good linear relationships were obtained in the respective linear ranges, with correlation coefficients (*R*^2^) no less than 0.990. The limits of detection (LODs) and quantification (LOQs) were 0.02-5 and 0.1-10 μg/kg, respectively. The recoveries of the 36 mycotoxins in fruits ranged from 77.0% to 118.9% at low, medium, and high spiked levels, with intra- and inter-day precisions in the range of 1.3%-14.9% and 0.2%-17.3%, respectively. The validated approach was employed to investigate mycotoxin contamination in actual fruit samples, including strawberry, grape, pear, and peach (15 samples of each type). Eleven mycotoxins, namely, altenuene (ALT), altenusin (ALS), alternariol-methyl ether (AME), tenuazonic acid (TeA), tentoxin (Ten), OTA, beauvericin (BEA), PAT, zearalanone (ZAN), T-2 toxin (T2), and mycophenolic acid (MPA), were found in the samples; three samples were contaminated with multiple mycotoxins. The incidence rates of mycotoxins in strawberry, grape, pear, and peach were 27%, 40%, 40%, and 33%, respectively. In particular, *Alternaria* toxins were the most frequently found mycotoxins in these fruits, with an incidence of 15%. The proposed method is simple, rapid, accurate, sensitive, reproducible, and stable; thus, it is suitable for the simultaneous detection of the 36 mycotoxins in different fruits.

水果是人们生活中必不可少的食物,其水分含量高、营养丰富,在生长、采摘、销售等环节易受到真菌的侵袭而产生真菌毒素^[1]^,每年约有20%~30%的水果会受到真菌毒素的污染而导致腐烂^[2]^。水果中常见的真菌毒素有链格孢霉毒素、赭曲霉毒素、展青霉素(PAT)、桔青霉素(CIT)和单端孢霉烯族毒素等^[3]^,大多具有致畸、致癌、致突变等毒性作用^[4]^。此外,即使已经去除腐烂水果的变质部分,在未腐烂部分也可能检出真菌毒素^[5]^,且真菌毒素具有较高的稳定性^[6]^,难以完全去除,给人类的健康造成潜在危害。我国《食品安全国家标准 食品中真菌毒素限量》(GB 2761-2017)规定,水果及其制品中PAT的限量为50 μg/kg,葡萄酒中赭曲霉毒素A(OTA)的限量为2.0 μg/kg,熟制坚果中黄曲霉毒素B_1_(AFB_1_)的限量为5 μg/kg^[7]^。欧盟也对果汁、果干和坚果等食品中PAT、OTA和AFB_1_的限量进行了规定^[8]^。因此,为确保食品质量安全,建立水果中真菌毒素的快速灵敏检测方法至关重要。

目前,真菌毒素的检测方法主要有超高效液相色谱-串联质谱法(UPLC-MS/MS)、液相色谱法、酶联免疫吸附测定法等,这些方法多针对单个或单类真菌毒素^[9⇓-11]^;其中,UPLC-MS/MS因其高选择性和高灵敏度在多种真菌毒素检测方面得到了广泛应用^[5,12]^,但其检测准确性及灵敏度受基质效应影响较大,需要通过有效的前处理过程去除基质中的大分子杂质和其他干扰因素。鲜食水果样品中主要含糖、色素和纤维素等杂质,其常见的前处理方法包括液液萃取(LLE)、固相萃取(SPE)和QuEChERS。LLE和SPE等方法成本较高,耗时费力,难以满足大批量样品的检测^[13,14]^,而QuEChERS因其快速、简便、安全、适用性广、提取净化效率高等优点,在真菌毒素检测方面具有较好的应用前景^[15⇓⇓-18]^。

本文主要针对草莓、葡萄、梨和桃4种水果基质,根据不同真菌毒素的物理化学性质,利用QuEChERS方法和UPLC-MS/MS检测技术的优势,建立了同时检测水果中36种真菌毒素的分析方法。本方法操作简单便捷,灵敏准确,能够为水果中多种真菌毒素的监测、监管提供有效技术手段。

## 1 实验部分

### 1.1 仪器、试剂与材料

UPLC XEVO TQ-S超高效液相色谱-串联质谱联用仪(美国Waters公司); 5804R离心机(德国Eppendorf公司); HSC-24B水浴氮吹仪(上海楚定分析仪器有限公司); Milli-Q超纯水仪(美国Millipore公司); AL104分析天平(美国梅特勒-托利多仪器有限公司); SK8210LHC超声波清洗机(上海科导超声仪器有限公司)。

甲醇、乙腈、醋酸铵(色谱纯,上海安谱实验科技股份有限公司); 36%乙酸(分析纯,上海阿拉丁生化科技有限公司);无水硫酸镁、氯化钠(分析纯,美国Sigma-Aldrich公司); 36种真菌毒素标准品见表1(青岛普瑞邦生物工程有限公司、ROMER国际贸易(北京)有限公司); *N*-丙基乙二胺(PSA, 50 μm)、十八烷基硅烷键合硅胶(C_18_,40~60 μm,苏州纳谱分析技术有限公司);实验所用草莓、葡萄、梨、桃均购自上海水果种植基地,水果经粉碎混匀后,装入密闭容器中,于-20 ℃下避光保存。

**表 1:** 36种真菌毒素的质谱参数

Mycotoxin	Abbreviation	Retention time/min	Precursor ion (m/z)	Product ions (m/z)	CEs/eV	Ionization mode
Patulin (展青霉素)	PAT	1.61	152.9	109.0^*^, 81.0	12, 14	-
Tenuazonic acid (细交链孢菌酮酸)	TeA	1.77	198.1	125.0^*^, 153.1	16, 12	+
Deoxynevalenol (脱氧雪腐镰刀菌烯醇)	DON	1.96	297.1	249.1^*^, 203.1	12, 14	+
Altenusin (细格菌素)	ALS	2.52	289.0	230.1^*^, 245.0	20, 17	-
Aflatoxin M_2_ (黄曲霉毒素M_2_)	AFM_2_	3.01	331.2	285.0^*^, 241.1	24, 42	+
15-Acetyl-deoxynivalenol (15-乙酰基-脱氧雪腐镰刀菌烯醇)	15-ADON	3.04	356.0	339.0^*^, 321.0	6, 12	+
3-Acetyl-deoxynivalenol (3-乙酰基-脱氧雪腐镰刀菌烯醇)	3-ADON	3.05	339.0	231.0^*^, 203.0	16, 16	+
Aflatoxin G_2_ (黄曲霉毒素G_2_)	AFG_2_	3.24	331.2	245.1^*^, 189.1	40, 30	+
Aflatoxin M_1_ (黄曲霉毒素M_1_)	AFM_1_	3.27	329.2	273.1^*^, 229.2	22, 38	+
Aflatoxin G_1_ (黄曲霉毒素G_1_)	AFG_1_	3.45	329.2	243.1^*^, 199.9	26, 42	+
Aflatoxin B_2_ (黄曲霉毒素B_2_)	AFB_2_	3.67	315.2	287.1^*^, 259.1	26, 28	+
Ochratoxin B (赭曲霉毒素B)	OTB	3.74	370.2	205.1^*^, 187.1	20, 34	+
Mycophenolic acid (麦考酚酸)	MPA	3.85	321.3	207.1^*^, 159.1	34, 20	+
Aflatoxin B_1_ (黄曲霉毒素B_1_)	AFB_1_	3.86	313.2	241.2^*^, 285.0	36, 22	+
Altenuene (交链孢霉烯)	ALT	3.92	293.0	257.1^*^, 275.1	14, 8	+
Diacetoxyscirpenol (蛇形毒素)	DAS	4.00	384.4	307.2^*^, 247.2	12, 10	+
Citrinin (桔青霉素)	CIT	4.00	251.0	233.1^*^, 205.1	16, 26	+
Ochratoxin A (赭曲霉毒素A)	OTA	4.36	404.3	239.0^*^, 221.0	22, 34	+
Alternariol (链格孢酚)	AOH	4.56	259.0	185.1^*^, 213.1	28, 24	+
HT-2 toxin (HT2毒素)	HT2	4.66	442.4	215.2^*^, 263.2	10, 10	+
β-Zearalanol (β-玉米赤霉醇)	β-ZAL	4.72	321.3	259.2^*^, 107.1	24, 36	-
Tentoxin (腾毒素)	Ten	4.82	415.2	312.2^*^, 199.2	18, 12	+
β-Zearalenol (β-玉米赤霉烯醇)	β-ZOL	4.88	319.3	160.0^*^, 174.2	26, 26	-
T-2 toxin (T2毒素)	T2	5.06	484.4	185.1^*^, 305.2	18, 14	+
α-Zearalanol (α-玉米赤霉醇)	α-ZAL	5.14	321.3	259.2^*^, 107.1	24, 36	-
α-Zearalenol (α-玉米赤霉烯醇)	α-ZOL	5.24	319.3	160.0^*^, 174.2	26, 26	-
Zearalanone (玉米赤霉酮)	ZAN	5.33	319.0	205.0^*^, 161.0	24, 24	-
Zearalenone (玉米赤霉烯酮)	ZEN	5.41	317.2	175.0^*^, 131.0	24, 26	-
Sterigmatocystin (杂色去霉素)	SMC	5.59	325.2	310.1^*^, 253.1	72, 72	+
Alternariol-methyl ether (交链孢霉甲基醚)	AME	5.73	273.0	258.0^*^, 128.1	25, 26	+
Ochratoxin C (赭曲霉毒素C)	OTC	5.75	432.1	239.0^*^, 358.1	24, 14	+
Enniatin B (恩镰孢菌素B)	ENNB	6.52	640.4	196.2^*^, 527.1	25, 22	+
Beauvericin (白僵菌素)	BEA	6.64	784.4	134.1^*^, 244.2	56, 30	+
Enniatin B_1_ (恩镰孢菌素B_1_)	ENNB_1_	6.70	655.6	196.0^*^, 210.0	23, 25	+
Enniatin A_1_ (恩镰孢菌素A_1_)	ENNA_1_	6.89	669.6	210.0^*^, 100.1	25, 40	+
Enniatin A (恩镰孢菌素A)	ENNA	7.10	682.5	210.4^*^, 328.1	25, 30	+

CEs: collision energies; * quantitative ion.

### 1.2 混合标准储备液的配制

分别移取适量的36种真菌毒素标准品原液,用乙腈稀释,配制成质量浓度为1 mg/L的36种真菌毒素混合标准储备液,混匀后保存于棕色试剂瓶内,置于-20 ℃冰箱中避光保存。根据实验需要再配制不同浓度的混合标准工作液,现配现用。

### 1.3 样品前处理

准确称取2.0 g试样于50 mL离心管中,加入10 mL乙酸-乙腈-水(1∶79∶20, v/v/v),涡旋混匀30 s后,超声提取40 min,加入2.0 g无水硫酸镁和0.5 g氯化钠,立即剧烈振摇30 s,超声10 min,以8000 r/min离心5 min;吸取6 mL上清液于含有85 mg C_18_、15 mg PSA的10 mL离心管中,涡旋振荡30 s,以8000 r/min离心5 min,移取5 mL上清液,于40 ℃下氮气吹干;用1 mL 5 mmol/L醋酸铵水溶液-乙腈(50∶50, v/v)溶解残渣,涡旋振荡30 s,经0.22 μm滤膜过滤后上机测定。

空白加标样品的前处理:选取经检测不含36种真菌毒素的4种空白样品(草莓、葡萄、梨、桃各1份),称取2.0 g试样于50 mL离心管中,加入一定体积的混合标准工作液后,按上述方法进行样品前处理。

### 1.4 基质混合标准溶液的配制

基质混合标准溶液:分别移取一定体积的36种真菌毒素混合标准储备液,再用空白基质提取液进行复溶和定容,配制成36种真菌毒素质量浓度分别为0.1、0.2、0.5、1、2、5、10、20、50、100、200 μg/L的系列基质混合标准溶液。

### 1.5 UPLC-MS/MS检测条件

色谱柱:Waters XBridge BEH C_18_柱(100 mm×3 mm, 2.5 μm);流动相A为5 mmol/L醋酸铵水溶液,流动相B为甲醇。梯度洗脱程序:0~0.2 min, 30%B; 0.2~5 min, 30%B~90%B; 5~7 min, 90%B; 7~7.5 min, 90%B~30%B; 7.5~8.5 min, 30%B。流速为0.4 mL/min;进样量为3 μL;柱温为40 ℃。

采用电喷雾电离源(ESI)正、负离子模式同时扫描;脱溶剂气、锥孔气均为高纯氮气,碰撞气为高纯氩气,脱溶剂温度为500 ℃,离子源温度为150 ℃;通过多反应监测(MRM)模式对目标化合物进行定量。36种真菌毒素的保留时间、母离子、子离子、碰撞能量等质谱参数见表1。分析数据使用MassLynxv4.1和Targetlynx(Waters)软件进行处理。

## 2 结果与讨论

### 2.1 色谱条件的优化

本实验考察了Waters XBridge BEH C_18_(100 mm×3 mm, 2.5 μm)、Waters Acquity UPLC BEH C_18_(100 mm×2.1 mm, 1.7 μm)、Agilent poroshell 120 EC-C_18_(100 mm×3 mm, 2.7 μm)和Waters Acquity UPLC HSS T3(100 mm×2.1 mm, 1.8 μm)4种不同型号、不同粒径色谱柱对36种真菌毒素的分离效果。结果发现,Waters XBridge BEH C_18_柱对36种真菌毒素的分离效果要明显优于其他3种色谱柱,36种真菌毒素不仅能在8.5 min内得到较好分离,且色谱峰形更加尖锐、对称,故选择Waters XBridge BEH C_18_柱(100 mm×3 mm, 2.5 μm)进行后续分析。

此外,还考察了5 mmol/L醋酸铵水溶液-甲醇、5 mmol/L醋酸铵水溶液-乙腈、0.1%甲酸水溶液-甲醇和0.1%甲酸水溶液-乙腈4组不同流动相体系对36种真菌毒素色谱峰峰形和分离的影响。实验表明,当采用5 mmol/L醋酸铵水溶液-乙腈作为流动相时,交链孢霉烯(ALT)、交链孢霉甲基醚(AME)、HT2毒素(HT2)、细交链孢菌酮酸(TeA)和腾毒素(Ten)等峰形较差;当采用0.1%甲酸水溶液-乙腈作为流动相时,AME、ALT、蛇形毒素(DAS)和TeA等峰形较差,CIT峰形拖尾严重,恩镰孢菌素B_1_(ENNB_1_)出峰滞后;当采用0.1%甲酸水溶液-甲醇作为流动相时,AME、CIT、DAS、ENNB_1_在峰形、响应和出峰时间上均有明显改善,但OTA、赭曲霉毒素B(OTB)峰形变差,且HT2出现双峰;当采用5 mmol/L醋酸铵水溶液-甲醇作为流动相时,36种真菌毒素的色谱峰形和响应强度最好,保留时间稳定。这是因为甲醇能更好地将样品中的目标物与干扰杂质分离^[19]^;且与0.1%甲酸水相比,在流动相中添加5 mmol/L醋酸铵更有利于不同目标物的离子化,既能保证良好的峰形又能增强大部分目标物的响应强度^[20]^。因此,最终选用5 mmol/L醋酸铵水溶液-甲醇作为流动相。

### 2.2 质谱条件的优化

通过单进样方式优化质谱条件,在ESI正、负离子模式下扫描(*m/z* 100~800),选择母离子。在确定母离子的基础上,进行子离子扫描,通过比较响应值选择2个子离子碎片。利用母离子的信号强度优化锥孔电压,子离子的信号强度优化碰撞电压,最终得到的母离子、子离子、锥孔电压和碰撞电压如表1所示。在最佳质谱条件的基础上建立了MRM扫描模式,对于同一个化合物,响应值高的通道作为定量离子通道,响应值略低的作为定性离子通道。36种真菌毒素混合标准工作液的MRM图谱如图1所示,不同水果样品基质中的MRM图谱如附图1~4所示(www.chrom-China.com),可见各目标毒素的峰形区分度良好。

**图 1:**
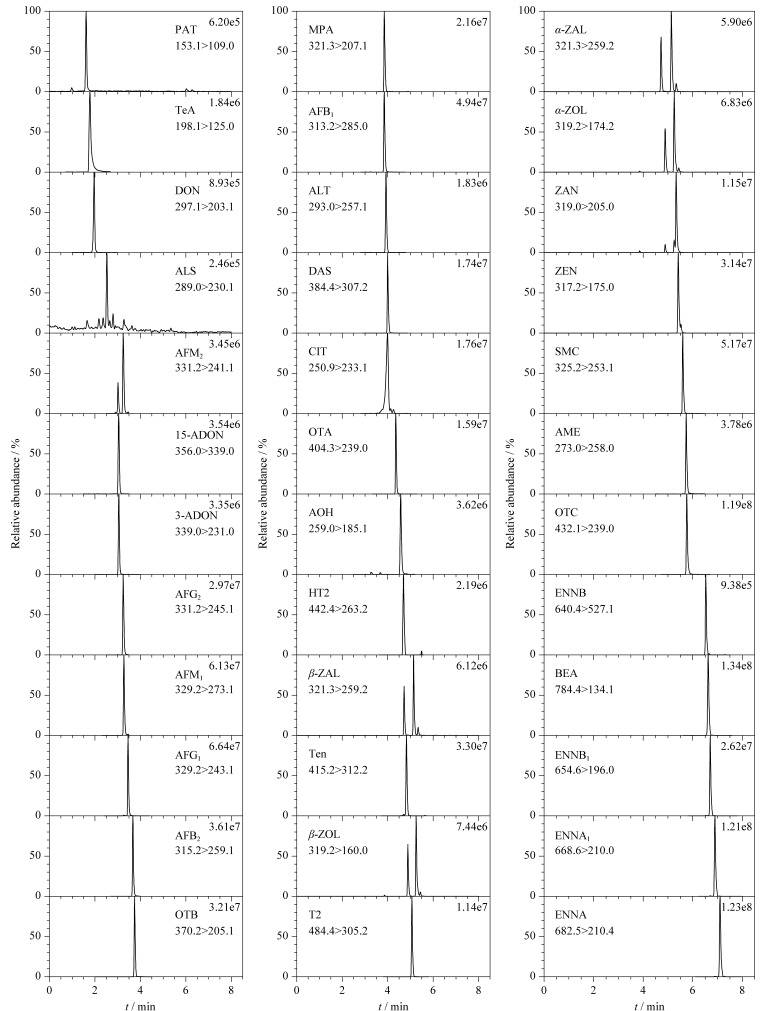
36种真菌毒素混合标准工作液(100 μg/L)的MRM色谱图

### 2.3 提取溶剂的优化

QuEChERS方法常用的提取溶剂为甲醇和乙腈,为了兼顾多种真菌毒素的检测,可适当添加一些酸或水来提高酸性毒素和极性毒素的提取效果。如图2所示,比较了甲酸-乙腈(1∶99, v/v)、甲酸-甲醇(1∶99, v/v)、甲酸-乙腈-水(1∶84∶15, v/v/v)、甲酸-乙腈-水(5∶80∶15, v/v/v)、乙腈-水(80∶20, v/v)和乙酸-乙腈-水(1∶79∶20, v/v/v)6种提取溶剂对36种真菌毒素(草莓空白加标样品,质量浓度50 μg/L)提取效果的影响。研究发现,甲酸-乙腈(1∶99, v/v)为提取溶剂时(图2a), 32种真菌毒素的回收率为70%~120%;甲酸-甲醇(1∶99, v/v)为提取溶剂时(图2b), 24种真菌毒素的回收率为70%~120%;以上结果表明,乙腈对真菌毒素具有更好的提取效果。在乙腈中加入不同比例的甲酸和水(图2c~2e),结果发现,乙腈-水(80∶20, v/v)作为提取溶剂时的提取效果优于甲酸-乙腈-水(1∶84∶15, v/v/v)、甲酸-乙腈-水(5∶80∶15, v/v/v),33种真菌毒素的回收率为70%~120%,但AFB_1_和黄曲霉毒素G_1_(AFG_1_)的回收率仅为36.1%和31.2%。为了提高AFB_1_和AFG_1_的回收率,将甲酸替换为酸性更弱的乙酸,发现乙酸-乙腈-水(1∶79∶20, v/v/v)提取溶剂对36种真菌毒素的提取效果最好(图2f),回收率均为70%~120%,这可能是由于黄曲霉毒素在弱酸中更稳定。因此选择乙酸-乙腈-水溶液(1∶79∶20, v/v/v)作为提取溶剂用于后续实验。

**图 2:**
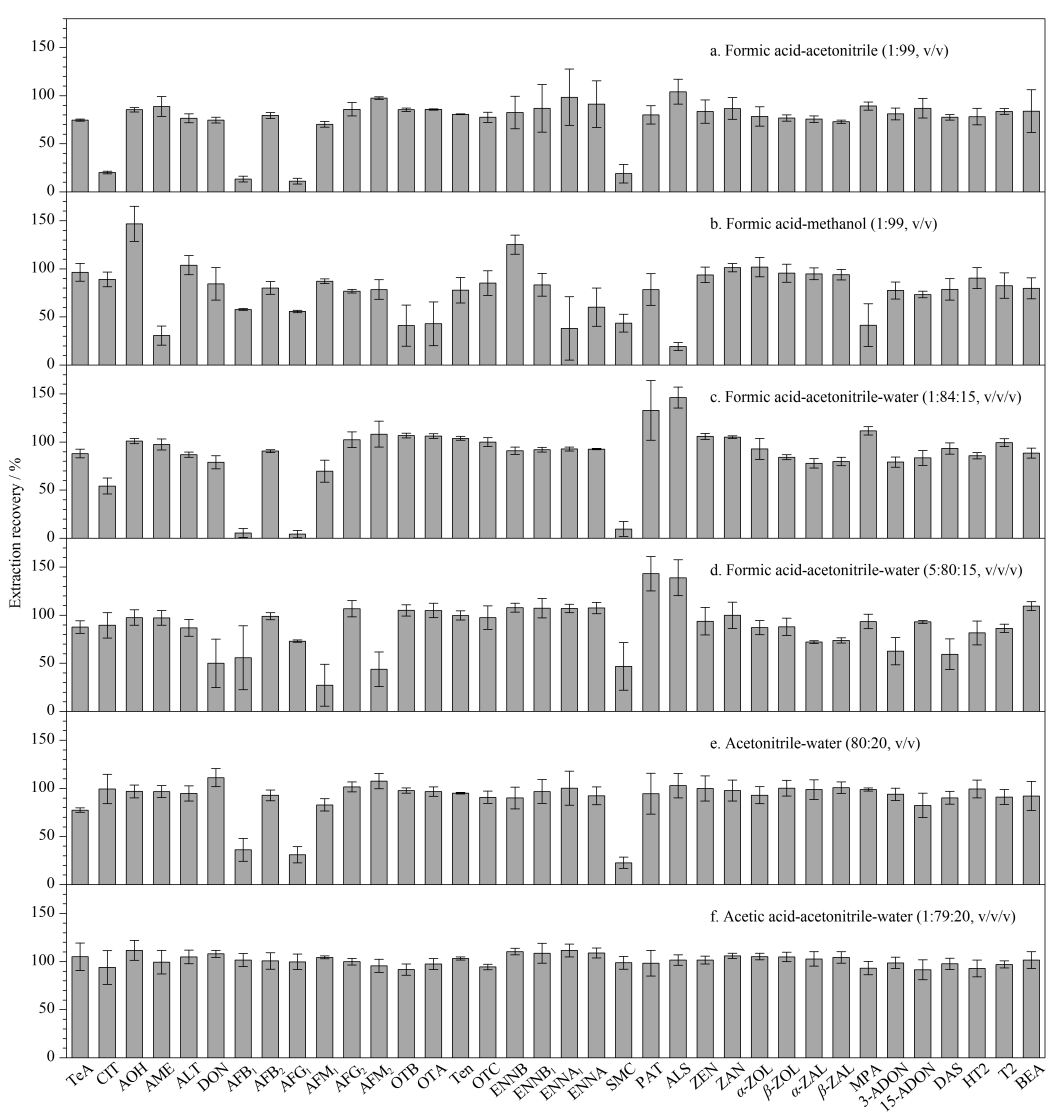
不同提取溶剂对36种真菌毒素提取回收率的影响(*n*=3)

### 2.4 净化材料的优化

在空白草莓样品中进行加标回收试验(加标质量浓度为50 μg/L),考察不同净化材料对36种真菌毒素回收率的影响。在未净化条件下,36种真菌毒素中有11种真菌毒素(脱氧雪腐镰刀菌烯醇(DON)、AFB_1_、AFG_1_、黄曲霉毒素M_1_(AFM_1_)、黄曲霉毒素M_2_(AFM_2_)、ENNB_1_、恩镰孢菌素A_1_(ENNA_1_)、恩镰孢菌素A(ENNA)、PAT、玉米赤霉烯酮(ZEN)和玉米赤霉酮(ZAN))的回收率较差,具有较大的基质效应,这是由于水果样品中含有大量的色素、有机酸、鞣质和纤维素等杂质,易造成色谱和质谱系统污染,并对目标物的检测产生较大干扰,因此需要对水果样品进行净化,降低基质效应(ME)^[21]^。常见的净化材料C_18_能有效去除脂肪和类脂等非极性杂质,PSA主要用于去除碳水化合物、酚类、脂肪和极性色素等,因此,本实验针对上述在未净化条件下回收率较差的11种真菌毒素,考察了不同组合比例的C_18_、PSA净化材料对真菌毒素回收率的影响(见图3)。研究发现,采用90 mg C_18_+10 mg PSA能够降低水果基质效应,除AFB_1_和AFG_1_的回收率仍大于120%外,其余9种真菌毒素的回收率为70%~120%;采用70 mg C_18_+30 mg PSA净化时,PAT的回收率大于120%,基质效应较强;而采用80 mg C_18_+20 mg PSA和85 mg C_18_+15 mg PSA均具有较好的净化效果,但采用85 mg C_18_+15 mg PSA时稳定性更好,36种真菌毒素的回收率为70%~120%。因此,最终选择85 mg C_18_+15 mg PSA作为净化材料对不同水果样品进行净化。

**图 3:**
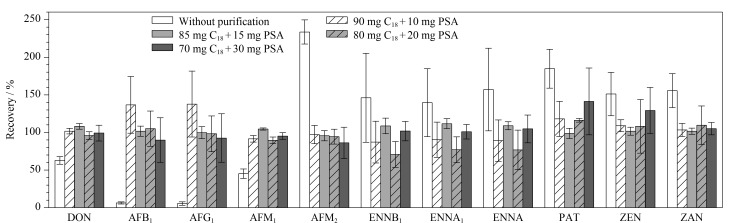
不同净化材料对11种真菌毒素回收率的影响(*n*=3)

### 2.5 方法学验证

#### 2.5.1 基质效应

通过考察36种真菌毒素在草莓、葡萄、梨和桃样品中的信号抑制/增强程度(signal suppression/enhancement, SSE)来评估基质效应^[22,23]^, SSE=*S*_1_/*S*_2_×100%,其中*S*_1_和*S*_2_分别为真菌毒素的基质标准曲线斜率和空白溶剂标准曲线斜率。当SSE大于100%,表明存在基质增强效应;SSE小于100%,表明存在基质抑制效应;SSE为80%~120%,表明基质效应影响较小。实验结果表明,部分真菌毒素如PAT、赭曲霉毒素C(OTC)和DON呈现基质抑制效应,SSE为3.6%~53.4%;少数真菌毒素如细格菌素(ALS)、ALT和AME呈现基质增强效应,SSE大于120%。因此,本研究采用空白基质匹配外标曲线法进行定量,以降低基质效应的影响。

#### 2.5.2 线性范围、检出限和定量限

利用空白基质提取液对36种真菌毒素的混合标准储备液进行稀释,得到不同质量浓度的系列基质标准溶液,以含量(*X*, μg/kg)为横坐标、峰面积(*Y*)为纵坐标,建立36种真菌毒素的基质匹配标准曲线;以3倍信噪比(signal to noise ratio, *S/N*)确定36种真菌毒素的检出限(limit of detection, LOD)、10倍信噪比确定定量限(limit of quantification, LOQ)。36种真菌毒素在不同水果样品中的线性范围、相关系数(*R*^2^)、LOD和LOQ如表2所示。36种真菌毒素在各自的线性范围内线性关系良好,*R*^2^均≥0.990; 36种真菌毒素在4种空白水果基质中的LOD和LOQ分别为0.02~5 μg/kg和0.1~10 μg/kg,满足《食品安全国家标准 食品中真菌毒素限量》(GB 2761-2017)等国家及行业相关限量标准的检测需求。

**表 2:** 36种真菌毒素在4种水果基质中的线性范围、相关系数、检出限和定量限

Mycotoxin	Strawberry						Grape						Pear						Peach							
	Linear range			LOD	LOQ		Linear range			LOD	LOQ		Linear range			LOD	LOQ		Linear range				LOD		LOQ	
PAT	10-	200	0.990	5	10		10-	200	0.994	5	10		5-	200	0.998	2	5		2-	200		0.996		1		2
TeA	5-	200	0.999	2	5		2-	200	0.997	1	2		5-	200	0.997	2	5		0.5-	200		0.998		0.2		0.5
DON	5-	200	0.999	2	5		5-	200	0.997	2	5		5-	200	0.992	2	5		5-	200		0.994		2		5
ALS	5-	200	0.999	2	5		0.1-	200	0.991	0.05	0.1		5-	200	0.998	2	5		1-	100		0.990		0.5		1
AFM_2_	0.5-	200	0.998	0.2	0.5		0.5-	200	0.996	0.2	0.5		2-	200	0.997	1	2		0.1-	200		0.995		0.04		0.1
15-ADON	5-	200	0.998	3	5		1-	200	0.996	0.5	1		5-	200	0.993	2	5		5-	200		0.995		3		5
3-ADON	2-	200	0.999	1	2		1-	200	0.996	0.6	1		5-	200	0.996	2	5		2-	200		0.999		1		2
AFG_2_	0.5-	200	0.996	0.1	0.5		0.5-	200	0.997	0.3	0.5		1-	200	0.997	0.5	1		0.5-	200		0.998		0.3		0.5
AFM_1_	0.5-	200	0.995	0.3	0.5		0.2-	200	0.994	0.1	0.2		1-	200	0.993	0.5	1		0.5-	200		0.994		0.3		0.5
AFG_1_	0.5-	200	0.999	0.2	0.5		0.1-	200	0.999	0.05	0.1		1-	200	0.993	0.4	1		0.5-	200		0.998		0.2		0.5
AFB_2_	0.1-	200	0.999	0.02	0.1		0.1-	200	0.999	0.05	0.1		1-	200	0.995	0.5	1		0.1-	200		0.995		0.05		0.1
AFB_1_	0.1-	200	0.992	0.05	0.1		0.2-	200	0.998	0.1	0.2		1-	200	0.992	0.5	1		0.1-	200		0.994		0.05		0.1
OTB	0.1-	200	0.999	0.05	0.1		1-	200	0.999	0.5	1		1-	200	0.998	0.5	1		1-	200		0.999		0.5		1
MPA	0.2-	200	0.995	0.05	0.2		0.2-	200	0.999	0.1	0.2		0.5-	200	0.997	0.3	0.5		0.5-	200		0.997		0.2		0.5
ALT	1-	200	0.999	0.5	1		0.5-	200	0.992	0.2	0.5		5-	200	0.994	3	5		2-	200		0.994		1		2
DAS	0.1-	200	0.999	0.05	0.1		0.1-	200	0.996	0.05	0.1		0.5-	200	0.999	0.3	0.5		0.2-	200		0.999		0.1		0.2
CIT	2-	200	0.999	1	2		1-	200	0.999	0.5	1		1-	200	0.996	0.5	1		2-	200		0.998		1		2
OTA	0.1-	200	0.999	0.02	0.1		0.1-	200	0.999	0.05	0.1		0.2-	200	0.998	0.1	0.2		0.1-	200		0.998		0.05		0.1
AOH	1-	200	0.999	0.2	1		2-	100	0.991	1	2		2-	200	0.996	2	5		2-	200		0.993		1		2
HT2	2-	200	0.999	1	2		2-	200	0.997	1	2		2-	200	0.999	1	2		1-	200		0.998		0.5		1
β-ZAL	1-	200	0.996	0.5	1		0.5-	200	0.999	0.3	0.5		1-	200	0.994	0.5	1		1-	200		0.996		0.6		1
Ten	0.1-	200	0.998	0.05	0.1		0.5-	200	0.991	0.3	0.5		0.5-	200	0.997	0.2	0.5		0.5-	200		0.996		0.2		0.5
β-ZOL	0.5-	200	0.999	0.2	0.5		0.5-	200	0.997	0.2	0.5		1-	100	0.995	0.6	1		1-	200		0.992		0.4		1
T2	0.1-	200	0.998	0.05	0.1		0.5-	200	0.991	0.3	0.5		0.1-	200	0.993	0.05	0.1		0.2-	200		0.998		0.1		0.2
α-ZAL	1-	200	0.998	0.5	1		0.5-	50	0.999	0.2	0.5		2-	200	0.991	1	2		1-	200		0.992		0.6		1
α-ZOL	0.5-	200	0.999	0.2	0.5		0.5-	50	0.998	0.2	0.5		1-	100	0.991	0.5	1		1-	100		0.997		0.5		1
ZAN	0.5-	200	0.997	0.2	0.5		0.5-	50	0.996	0.2	0.5		0.2-	100	0.992	0.1	0.2		1-	100		0.996		0.5		1
ZEN	1-	200	0.996	0.5	1		0.1-	50	0.997	0.05	0.1		0.5-	200	0.991	0.2	0.5		1-	100		0.995		0.5		1
SMC	0.1-	50	0.999	0.05	0.1		1-	200	0.994	0.5	1		0.1-	100	0.997	0.05	0.1		0.5-	100		0.993		0.2		0.5
AME	1-	200	0.999	0.5	1		2-	100	0.990	1	2		5-	200	0.990	3	5		1-	200		0.991		0.5		1
OTC	0.1-	100	0.995	0.05	0.1		0.5-	50	0.997	0.2	0.5		0.2-	100	0.993	0.1	0.2		1-	200		0.994		0.5		1
ENNB	0.1-	200	0.999	0.05	0.1		0.1-	50	0.996	0.04	0.1		0.5-	100	0.992	0.2	0.5		1-	200		0.992		0.5		1
BEA	0.1-	200	0.996	0.05	0.1		1-	200	0.993	0.5	1		2-	200	0.998	1	2		0.1-	200		0.992		0.05		0.1
ENNB_1_	0.1-	200	0.999	0.05	0.1		0.1-	50	0.998	0.05	0.1		0.5-	100	0.991	0.2	0.5		0.1-	200		0.992		0.05		0.1
ENNA_1_	0.1-	200	0.999	0.02	0.1		0.1-	50	0.995	0.05	0.1		1-	100	0.992	0.5	1		0.1-	200		0.992		0.05		0.1
ENNA	0.1-	200	0.999	0.05	0.1		0.2-	50	0.997	0.1	0.2		1-	100	0.991	0.5	1		0.1-	200		0.992		0.06		0.1

*: unitless.

#### 2.5.3 回收率和精密度

按照1.3节方法处理样品,并进行加标回收和精密度试验,在4种空白水果基质中分别添加低、中、高(5、50、100 μg/L)3个不同水平的36种真菌毒素混合标准工作液,根据测定值和理论值的比值计算各自的回收率;日内精密度和日间精密度分别为同一天5次平行试验和不同自然日(*n*=3)测定结果的相对标准偏差(relative standard deviation, RSD)。如附表1~4所示,36种真菌毒素在草莓样品中的平均回收率为83.8%~118.9%,日内精密度为2.0%~13.9%,日间精密度为0.2%~17.3%;在葡萄样品中的平均回收率为77.0%~118.8%,日内精密度为1.7%~14.9%,日间精密度为0.3%~14.9%;在梨样品中的平均回收率为81.7%~116.3%,日内精密度为2.4%~14.8%,日间精密度为1.2%~14.9%;在桃样品中的平均回收率为81.1%~118.6%,日内精密度为1.3%~14.9%,日间精密度为0.3%~14.9%。以上结果表明,所建立的真菌毒素高通量检测方法准确可靠,可用于草莓、葡萄、梨和桃等实际样品的分析。

#### 2.5.4 方法比较

为了考察本方法的适用性,将其与已报道相关研究进行对比,结果如表3所示。本实验所建立的QuEChERS-UPLC-MS/MS方法覆盖真菌毒素数量多,C_18_和PSA净化材料用量少,检测时间短,线性范围宽,灵敏度高,可用于草莓、葡萄、梨和桃等水果样品中36种真菌毒素的同时高通量检测。

**表 3:** 与已报道文献中真菌毒素检测方法的比较

Samples	Numbers of myco- toxins	Extraction solvents	Purification materials	Mobile phases	Linear range/ (μg/kg)	LODs and LOQs/ (μg/kg)	Ref.
Maize	20	acetonitrile-water-formic acid (80∶18∶2, v/v/v)	300 mg magnesium sulfate, 150 mg PSA and 150 mg C_18_	0.1% formic acid aqueous solution, methanol	0.25-200	0.17-6.67, 0.5-20	[19]
Nutmeg	21	acetonitrile-water-formic acid (84∶15∶1, v/v/v)	100 mg C_18_ and 200 mg magnesium sulfate	0.1% formic acid methanol, 2 mmol/L ammonium formate aqueous solution	0.125-1000	0.04-3.5, 0.125-10	[24]
Tea	6	acetonitrile-water-acetic acid (79∶20∶1, v/v/v)	150 mg C_18_ and 150 mg PSA	5 mmol/L ammonium formate aqueous solution, 5 mmol/L ammonium formate methanol	-	1, 1	[25]
Ketchup	5	acetonitrile, water	0.5 g magnesium sulfate and 100 mg C_18_	0.1% formic acid aqueous solution, acetonitrile	0.5-200	1, 2.5	[26]
Nut	20	acetonitrile	0.2 g C_18_	acetonitrile, 0.05% formic acid aqueous solution	1-500	0.03-2.25, 0.11-7.50	[27]
Tomato	9	acetonitrile-water-formic acid (84∶15∶1, v/v/v)	200 mg C_18_	5 mmol/L ammonium acetate aqueous solution, methanol	1-200	0.3-1.5, 1-10	[28]
Fruits	36	acetic acid-acetonitrile- water (1∶79∶20, v/v/v)	85 mg C_18_ and 15 mg PSA	5 mmol/L ammonium acetate aqueous solution, methanol	0.1-200	0.02-5, 0.1-10	this study

-: not mentioned.

### 2.6 实际样品的检测

利用所建立方法对上海不同地区的60份水果样品(草莓、葡萄、梨和桃各15份)进行36种真菌毒素的测定。结果如表4所示,共有21份(35%)水果样品检出真菌毒素,包括PAT、OTA、ZAN、T2毒素(T2)、ALT、ALS、白僵菌素(BEA)、TeA、Ten、AME和麦考酚酸(MPA)等11种真菌毒素,平均含量为0.13~35.85 μg/kg。草莓、葡萄、梨和桃4种基质中真菌毒素的检出率分别为27%、40%、40%和33%,其中在草莓样品中检出ALT和PAT两种真菌毒素,检出率分别为7%和27%,平均含量为20.52~35.85 μg/kg;在葡萄样品中检出AME、BEA、MPA、PAT、Ten 5种真菌毒素,检出率为7%~20%,平均含量为0.96~6.06 μg/kg;在梨样品中检出TeA、OTA、T2、BEA、ZAN和ALS 6种真菌毒素,检出率为7%~13%,平均含量为0.20~18.65 μg/kg;在桃样品中,共检出TeA、AME、OTA、T2和BEA 5种真菌毒素,检出率均为7%,平均含量为0.13~11.77 μg/kg。有3份(5%)水果样品同时检出两种或两种以上的真菌毒素,在1份草莓样品中同时检出ALT和PAT, 1份葡萄样品中同时检出AME、BEA和PAT, 1份梨样品中同时检出TeA和ZAN。此外,实验发现不同水果中链格孢毒素(ALT、ALS、TeA、Ten、AME)的检出率较高,检出率为15%。以上检测结果表明,水果在生长、采摘、销售等环节可能受到多种真菌侵染而产生真菌毒素,实际样品中检出的OTA和PAT含量虽均低于国家限量标准,但仍存在一定风险隐患,需引起重视。

**表 4:** 实际水果样品中真菌毒素的检测结果

Mycotoxin	Strawberry			Grape			Pear			Peach	
	Detection rate/%	Average content/ (μg/kg)		Detection rate/%	Average content/ (μg/kg)		Detection rate/%	Average content/ (μg/kg)		Detection rate/%	Average content/ (μg/kg)
ALS	-	-		-	-		13	18.65		-	-
ALT	7	35.85		-	-		-	-		-	-
AME	-	-		7	2.17		-	-		7	8.08
BEA	-	-		20	2.04		7	2.09		7	0.19
MPA	-	-		7	3.30		-	-		-	-
OTA	-	-		-	-		7	0.20		7	0.13
PAT	27	20.52		7	6.06		-	-		-	-
T2	-	-		-	-		7	2.58		7	0.45
TeA	-	-		-	-		7	6.00		7	11.77
Ten	-	-		13	0.96		-	-		-	-
ZAN	-	-		-	-		7	7.00		-	-

-: not detected.

## 3 结论

本研究基于QuEChERS前处理方法和UPLC-MS/MS检测技术,建立了同时检测草莓、葡萄、梨和桃等水果中36种真菌毒素的方法。实验中对色谱、质谱、提取溶剂、净化材料等条件进行了优化,有效减少了基质干扰,提高了检测灵敏度和回收率。本方法操作简单,检测时间短,覆盖毒素数量多,线性范围宽,准确度和精密度高,能够满足国家和行业标准的相关检测要求,为水果中真菌毒素的污染监测提供了检测依据和技术参考。实际水果样品检测发现,35%的样品检测出真菌毒素,其中5%的样品检测出两种及两种以上真菌毒素,且链格孢毒素污染较为严重。因此,鲜食水果在采收、销售等环节中仍存在真菌毒素侵染的风险隐患,需要加强监测和监管。
